# Atmospheric Pressure Photoionization with Halogen Anion Attachment for Mass Spectrometric Analysis of Hydrocarbons and Hydrocarbon-Based Polymers

**DOI:** 10.1021/jasms.4c00331

**Published:** 2024-11-05

**Authors:** Essi Rytkönen, Juha Rouvinen, Janne Jänis, Marko Mäkinen

**Affiliations:** Department of Chemistry, University of Eastern Finland, P.O. Box 111, FI-80101 Joensuu, Finland

## Abstract

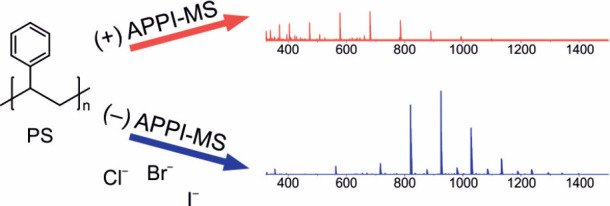

Aliphatic hydrocarbons and hydrocarbon-based synthetic polymers are of interest in many fields, but their characterization by mass spectrometric methods is generally limited due to their poor ionizability. Recently, atmospheric pressure photoionization (APPI), combined with halogen anion attachment in negative-ion mode, has drawn attention as a potential method for ionizing various polymers without extensive fragmentation or other unwanted side reactions. In this work, the applicability of halogen anion attachment with APPI was studied using several synthetic polymers, including polyethylene, polypropylene, polyisoprene, and polystyrene, as well as simple *n*-alkanes of various chain lengths. For hydrocarbon-based polymers, the method produced clear distributions of intact polymer adduct ions when different halogen anions were used. It was found that increasing the halogen anion size decreased ionization efficiency, particularly in the absence of π-bonds in the polymer structure. Testing with simple *n*-alkanes showed that only molecules containing fifty or more carbon atoms formed detectable halogen adducts, possibly due to the low gas-phase stabilities of the lighter *n*-alkane adduct ions. In conclusion, halogen anion attachment with negative-ion APPI appears to be a highly promising method for polymer analysis, providing structural data and clean polymer mass spectra with minimal fragmentation, which can be useful for the identification of unknown samples.

## Introduction

Synthetic polymers are widely used materials due to their many advantageous properties, such as durability, lightweight, low production cost, and safety, and thus they have become essential for everyday life.^1,2^ A majority of plastic market consists of hydrocarbon-based polymers such as polyethylene (PE) and polypropylene (PP).^3,4^ These polymers are especially resistant to biodegradation, leading to their accumulation in nature and their harmful effects to the ecosystems.^2,5,6^ Currently, plastic polymers are recognized as a global pollution problem, and many efforts have been made to find solutions for reducing plastic waste.^1,2,7^ Because of the increasing interest in polymer recycling and degradation, straightforward analysis techniques to characterize physicochemical changes occurring in these processes are highly desired.

One of the key techniques for polymer analysis is mass spectrometry, which provides detailed information about polymer branching, composition of building blocks, polymer end groups, and molecular weight (MW) distributions.^8−10^ Polymer analysis by mass spectrometry generally utilizes soft ionization methods such as matrix-assisted laser desorption/ionization (MALDI) or electrospray ionization (ESI).^8,11,12^ However, hydrocarbon-based polymers generally ionize poorly with these methods since they lack easily ionizable (acidic or basic) functional groups.^13−16^ In recent years, atmospheric pressure photoionization (APPI) has gained more attention in polymer analysis. APPI can ionize nonpolar compounds through a series of reactions initiated by the vacuum ultraviolet (VUV) photons emitted from the krypton discharge lamp.^17,18^ The main ionization mechanisms include direct photoionization or solvent/dopant-mediated proton transfer, but anion attachment is also a possible route to ion formation in the negative-ion mode.^18,19^ The latter has been utilized in a few recent studies, where polymers have been characterized as their negative halogen adduct ions.^12,13,20^

The first studies adapting halogen anion attachment to the polymer analysis were conducted by Kéki et al.^13,20^ who focused on two hydrocarbon-based polymers, polyethylene and polyisobutylene (PIB). Both polymer types formed abundant [M + Cl]^−^ ions of intact PE and PIB oligomers in negative-ion APPI with the addition of a chlorinated solvent (CCl_4_, CHCl_3_, or CH_2_Cl_2_), whereas positive-ion APPI led to the formation of protonated molecules with extensive polymer chain fragmentation, resulting in underestimated average molar mass and polydispersity values. A detailed mechanism for the adduct ion formation was also proposed, starting with a direct photoionization of the toluene dopant, followed by a dissociative electron capture of the halogenated solvent and the gas-phase adduct formation between the donated halogen anions and the polymer.^13,20^ Both PE and PIB required the use of dopant (toluene) for efficient ionization.

The proposed mechanism was further confirmed by Desmazières et al.,^12^ who focused on halogen adduct formation of different polymers with varying photon energies and solvents. The experiments demonstrated that the polymer ionization is only dependent on the dopant and not the polymer or the solvent, thus verifying that the adduct formation begins with the dopant ionization. In addition, they showed that the ionization of polystyrene and other synthetic polymers is possible with halogen attachment, and clear distributions of halogen adduct ions are produced.^12^ The method has also been adapted to the analysis of labile end groups in polystyrene and was deemed suitable for characterization.^21^ Lastly, halogen anion attachment has been used to study polyalphaolefins (PAOs), for which halogen adduct formation allowed for the characterization of polymer repeating units and, consequently, the monomers used in the polymer synthesis.^22^ They also found that low-viscosity grade PAOs (∼C_30_–C_50_) did not form any halogen adducts.

The above-mentioned studies have confirmed the suitability of halogen ion attachment for polymer analysis, but the possibility to extend this method to the analysis of short hydrocarbons is also very interesting as hydrocarbon ionization would be desirable in many applications such as petroleomics.^23,24^ Saturated hydrocarbons are challenging to ionize since they often give similar fragments with more energetic methods (e.g., electron ionization) or react with the bath gas to make oxygen or nitrogen “adducts”.^23^ Thus, information about their structure and molecular weight is not easily obtained.^25^ Several different ionization methods have been explored to analyze large hydrocarbons in complex mixtures, including atmospheric pressure chemical ionization (APCI),^23,24,26^ field desorption/ionization (FD/FI),^27^ laser-induced acoustic desorption–chemical ionization (LIAD–CI)^28^ and nitrogen insertion.^29^ Still, alternative techniques are studied to find a broadly applicable method for hydrocarbon ionization.

**Figure 1:**
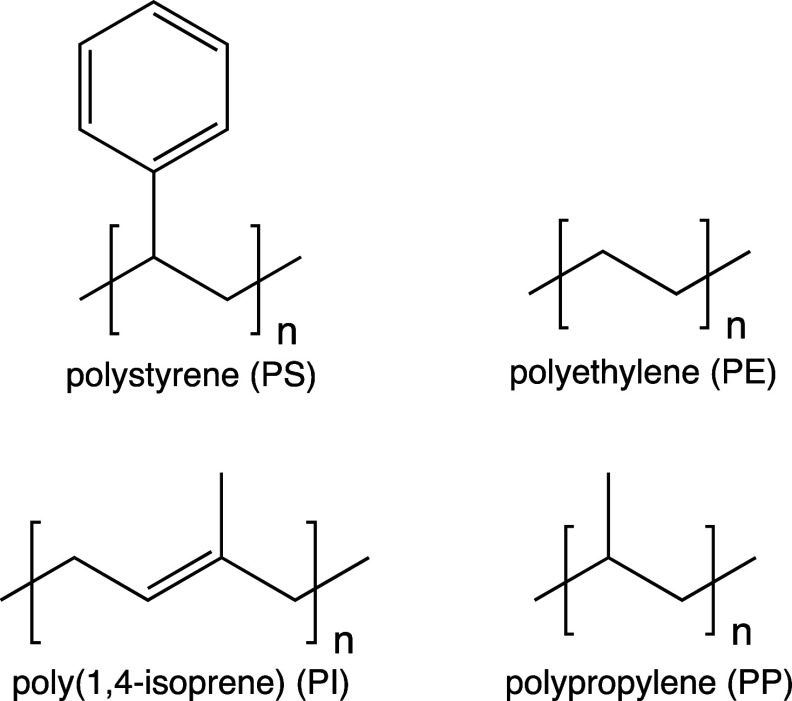
Structures of the polymers studied in this work.

The main aim of this work was to further study halogen anion adduct formation in negative-ion APPI for hydrocarbon-based polymers (Figure 1) and compare that to the conventional positive-ion APCI/APPI. The studied polymers included polyethylene, polypropylene, polyisoprene, and various polystyrene samples. Three halogen anions (Cl^–^, Br^–^, and I^–^) were chosen to compare their efficiency in the adduct ion formation with different polymers materials. Moreover, *n*-alkanes with different carbon chain lengths were also studied to establish the suitability of the chosen method for hydrocarbon analysis.

## Experimental Section

### Materials

Four hydrocarbon-based polymers with varying molar masses were characterized in this study. All polystyrene samples (PS162, PS266, PS370, PS560, PS1000, and PS1800), as well as one polyethylene (PE800) and two poly(1,4-isoprene) standards (PI800 and PI1200), were purchased from PSS Polymer Standards Service GmbH (Mainz, Germany). Another PE standard (PE4000) as well as isotactic (iPP) and amorphous (aPP) polypropylene samples were purchased from Sigma-Aldrich (St. Louis, MO). The molecular masses of all the polymer standards (based on the supplier data) are presented in Table S1.

In addition to polymers, various *n*-alkanes with chain lengths from 14 to 60 carbon atoms were obtained from different suppliers: tetradecane and pentadecane from Alfa Aesar (Haverhill, MA), hexadecane from Fischer Chemicals (Waltham, MA), octadecane from Acros Organics (Geel, Belgium), heneicosane, pentacosane, triacontane, tetracontane from Sigma-Aldrich, and pentacontane and hexacontane from Supelco (Bellefonte, PA). In addition, two hydrocarbon standards (i.e., *n*-alkane mixture of C_21_–C_40_ and a hydrocarbon mixture of C_10_, C_16_, C_34_, and C_50_) were purchased from Sigma-Aldrich. The solvents used in the experiments (toluene, dichloromethane, dibromomethane, diiodomethane, and chloroform; HPLC grade) were obtained from VWR Chemicals (VWR International Oy, Helsinki, Finland).

### Sample Preparation

All polymer standards were dissolved in toluene to a concentration of 5 mg/mL. In the case of PE and PP, solutions were ultrasonicated to increase the amount of the dissolved polymer. For (+) APPI, the samples were prepared by diluting the stock solutions to the concentration of 0.5 mg/mL with toluene. For (−) APPI, the samples were prepared by diluting the polymer stock solutions to a concentration of 0.5 mg/mL and adding 10 vol % of either CH_2_Cl_2_, CH_2_Br_2_, or CH_2_I_2_ to form halogenated adduct ions. Due to the low solubility of isotactic polypropylene to toluene, the stock solution was ultrasonicated for one hour, after which the mass spectrometry sample was prepared with 1:10 (v/v) dilution to toluene, even though the polymer was not completely dissolved by visual inspection. Similar to other samples, 10 vol % of halogenated solvent was added for (−) APPI analysis.

Hydrocarbon stock solutions were prepared by dissolving the samples in toluene to the concentration of 1 or 5 mg/mL. The solutions were ultrasonicated in situations where the *n*-alkane had low solubility in toluene. The samples were prepared as in the case of polymers, i.e., stock solutions were diluted with toluene to the concentration of 0.5 mg/mL and 10 vol % of CH_2_Cl_2_ was added. Other solvents, such as chloroform, were also used to find the optimal solvent for alkane solubility.

### Mass Spectrometry

All mass spectra were collected on a Bruker timsTOF quadrupole time-of-flight (Q-TOF) instrument (Bruker Daltonics GmbH, Bremen, Germany), fitted with a standard Bruker APPI or APCI ion source. The mass resolving power was ∼30,000 (fwhm) at *m*/*z* 600 and the obtained RMS mass error was ∼0.1 ppm, across the measured mass spectra. The vaporizer temperature was set to 400 °C, and the capillary voltage was 1500 V with the end plate offset of 500 V. Nitrogen was used both as the drying (200 °C, 4.0 L/min) and nebulizing gas (2 bar). The sample solutions were directly introduced to the ion source via a syringe pump operated at a flow rate of 300 μL/h. The mass spectra were recorded in the *m*/*z* range of 50–3000. Bruker otofControl 5.1 software was used for the instrument operation and data acquisition. The instrument was calibrated externally using an APCI-L low concentration tuning mix (Agilent Technologies, Santa Clara, CA, USA). The acquired mass spectra were further internally recalibrated using selected analyte ions. The data postprocessing and analysis was accomplished using Bruker DataAnalysis 5.1 software.

### Calculation of Molecular Weights

The number average molar weight (*M*_n_), the weight average molecular weight (*M*_w_), and the polydispersity (*D*_M_) were calculated for the analyzed polymer samples using eqs 1–3,
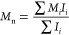
1
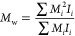
2
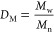
3where *M*_i_ = *m*/*z* of the ion and *I*_i_ = absolute intensity of the ion. All the observed polymer (adduct) ions were included in the calculations. Peak molecular weight values (*M*_p_) were also used for comparison. The mass of the halogen anion was subtracted from the calculated *M*_n_, *M*_w_, and **D**_M_ values.

## Results and Discussion

In order to find a suitable starting point for polymer analysis, preliminary experiments were conducted using positive-ion APCI or APPI. These ionization methods were observed to cause extensive fragmentation and also result in the formation of oxygenated species, possibly via reactive oxygen species (like superoxide radicals, O_2_^–•^) formed in the ion source. On the basis of these preliminary experiments and the literature, negative-ion APPI with halogen anion attachment was chosen for further studies, since it was found to produce “clean” polymer ion distributions with minimal fragmentation. Additionally, chlorinated solvent (CH_2_Cl_2_) was found to produce mass spectra with the highest signal-to-noise ratios, and thus it was mostly used in the experiments for halogen adduct formation.

**Figure 2:**
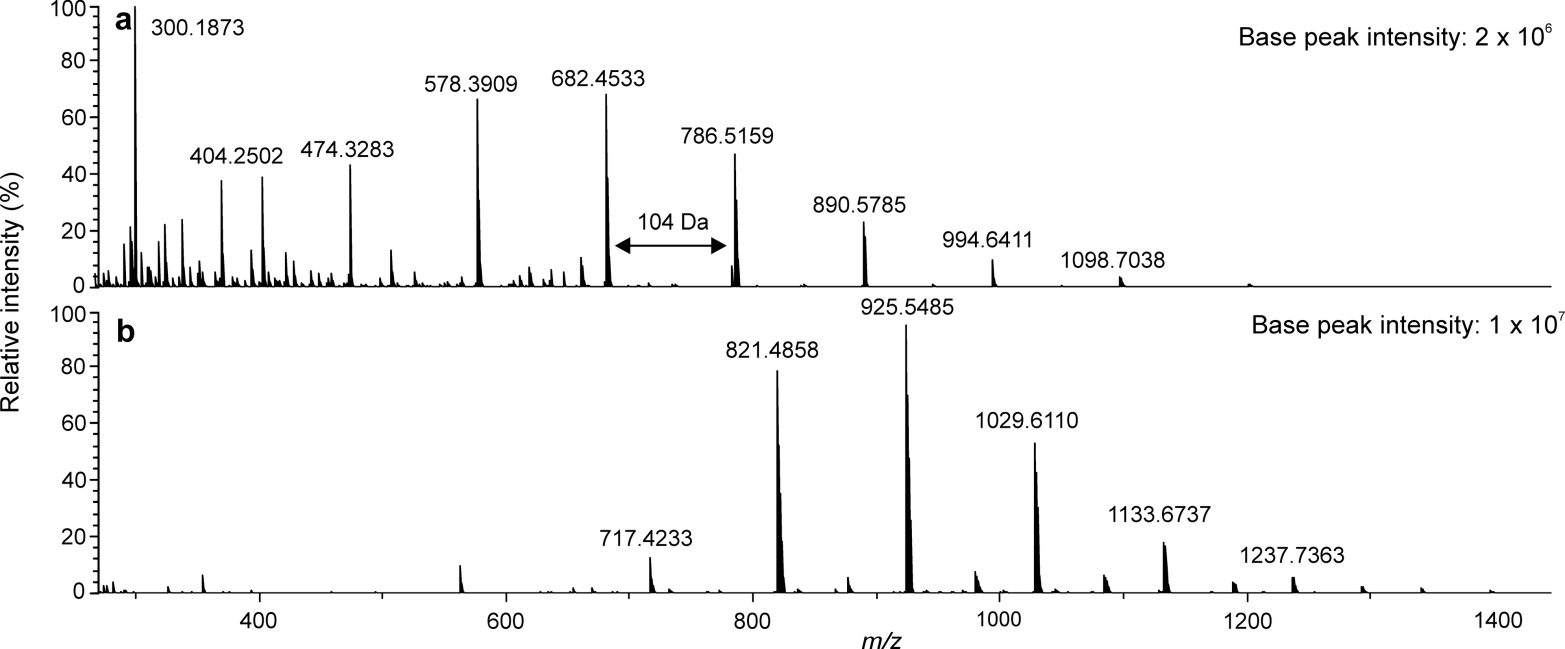
APPI-TOF mass spectra for PS560 in (a) positive-ion mode with toluene as the solvent and (b) negative-ion mode with toluene as the solvent and CH_2_Cl_2_ for halogen adduct formation. The monoisotopic *m*/*z* values of the ions have been given. The absolute base peak intensities are reported for sensitivity comparison.

Halogen anion attachment was deemed a feasible method for polymer analysis, but the average molecular weights obtained from the mass spectra were under- or overestimated when compared to the reported values (Table S1). Overestimation could be due to lower halogen anion binding energies of the smallest oligomers, which decreases gas-phase stabilities of the formed adduct ions.^22^Figure 2 depicts the mass spectra obtained for PS560 sample by (+) APPI and (−) APPI with CH_2_Cl_2_ as the halogen anion donor. With (+) APPI, low-mass fragment ions were clearly observed in the mass spectrum, in addition to intact radical cations of the PS oligomers, separated by 104 Da (i.e., styrene monomer). In contrast, (−) APPI did not show signals for the smallest PS oligomers in the sample. The average MW of PS560 (Table S1) is lower than the mass of *n*-pentacontane which formed the smallest detected adduct ions. It is therefore apparent that there is a severe discrimination toward the smallest oligomers and thus on overestimation of the average MW (i.e., the distribution peaking at *m*/*z* 926). The calculated polydispersity for PS560 was higher with (+) APPI (*D*_M_ = 1.12) than with (−) APPI (*D*_M_ = 1.01), likely because of the low mass discrimination as well.

In the earlier study by Desmazières et al., it was also reported that heavier polymer chains likely decompose^12^ and cannot be detected. Optimal detection of polymer ion distribution is also dependent on the ion source/desolvation and ion transfer region conditions, such as the source temperature.^12^ Thus, the observed MW distribution for a given polymer may be under- or overestimated in the APCI/APPI-MS analysis depending on the conditions. While (−) APPI with halogen ion adduction may not lead to accurate molecular weight estimations, it has many advantages over the other methods. For example, for PS560 sample, a 5-fold base peak intensity was observed with (−) APPI as compared to (+) APPI (Figure 2). Moreover, (−) APPI caused less fragmentation and produced much less background noise. Furthermore, undesired oxygenation is also absent.

### Polystyrene

**Figure 3:**
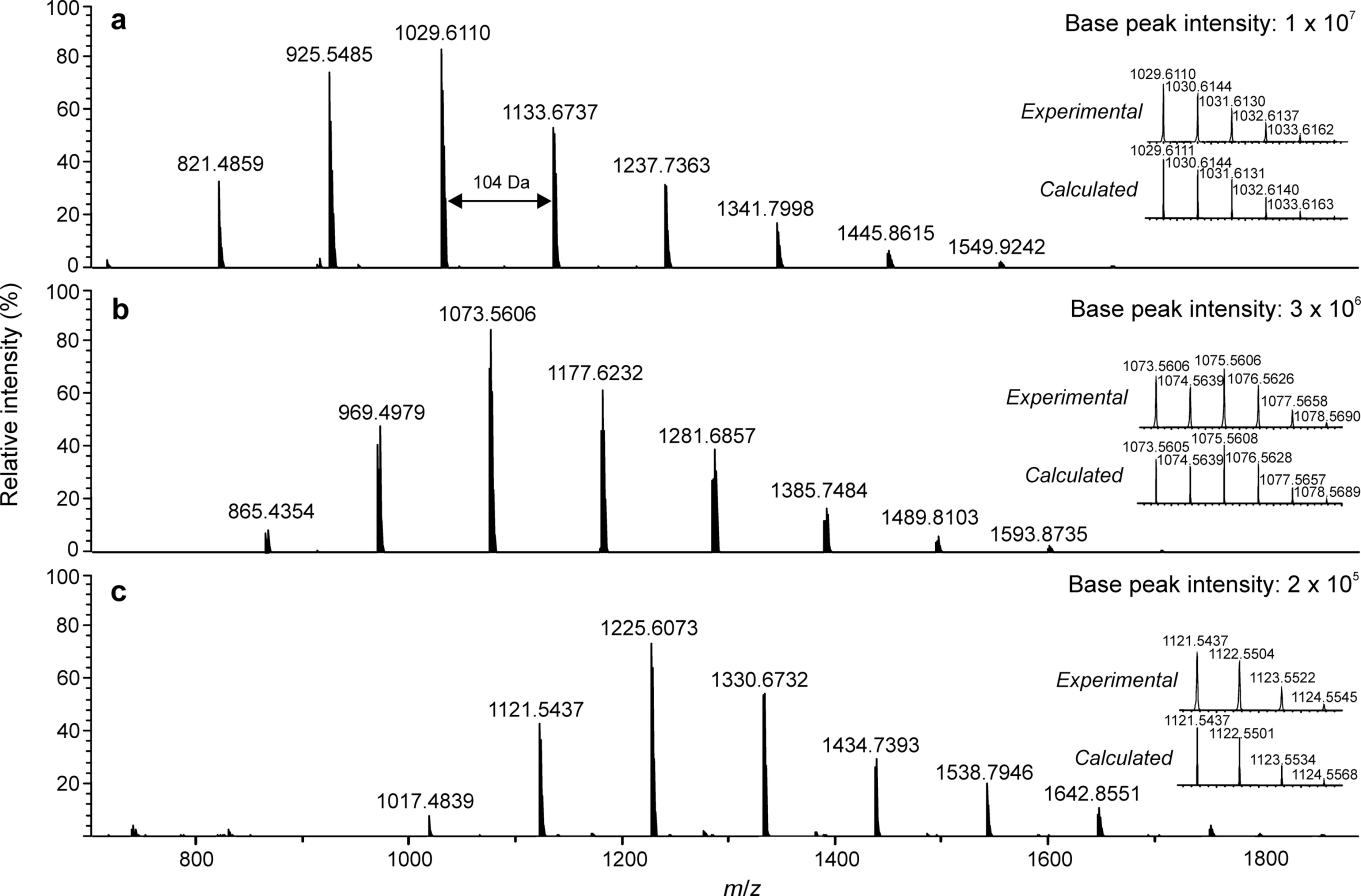
Negative-ion APPI-TOF mass spectra for PS1000 with (a) CH_2_Cl_2_, (b) CH_2_Br_2_, or (c) CH_2_I_2_ as the halogenated solvent and toluene as the dopant. The insets show the comparisons between the experimental and theoretical isotopic distributions for the PS oligomer *n* = 9 ([C_76_H_82_ + X]^−^) in all cases. The absolute base peak intensities are reported for sensitivity comparison.

Positive- and negative-ion APPI-MS analyses of polystyrene samples resulted in differences in the molecular weight distributions. In the positive-ion mode, polystyrene oligomers fragmented into smaller oligomers, causing molecular weight distributions to shift to lower *m*/*z* values. The calculated *M*_n_ values with (+) APPI for PS560, PS1000, and PS1800 were 591, 805, and 1211, and the corresponding *M*_w_ values were 659, 889, and 1291, respectively. Several ion distributions, including molecular ions (M^+•^) and fragment ions, could be detected. In contrast, negative-ion mode combined with halogen anion attachment resulted in the single [M + X]^−^ (X = Cl, Br, or I) ion series with minimal fragmentation and background noise. For PS1000 (Figure 3), the calculated average MWs were close to the reference values or slightly overestimated (iodide). On the contrary, for PS1800 (Figure S1), molecular weights were clearly underestimated (e.g., for the chloride adducts, *M*_n_ = 1268 and *M*_w_ = 1302), probably due to halide losses from the heavier oligomers, as discussed earlier. Nevertheless, halogen adduct formation with (−) APPI was found advantageous as compared to (+) APPI, since it enabled the characterization of polystyrene with minimal fragmentation.

Halogen adducts in each case were confirmed by comparison of their experimental and simulated isotopic patterns (Figures 3 and S4). Accurate masses provided information about the polymer composition, for example ion at *m*/*z* 1029.6110 (Figure 3a) corresponds to the PS oligomer with 9 repeating units as well as butyl and hydrogen end groups,^21^ consistent with the molecular formula of [C_76_H_82_ + Cl]^−^. The mass difference between the adjacent peaks was 104 Da, as expected for PS with a repeating unit of C_8_H_8_, and a few additional peaks indicating minimal fragmentation. The results are consistent with the previous reports of chloride and bromide attachment.^12,21^ Iodide was also capable of forming halogen adducts with polystyrene.

Small PS oligomer standards (PS162, PS266, and PS370) ionized with (+) APPI, but no halogen adducts were detected with (−) APPI instead. This observation could be due to low stability of the adducts in the gas phase, which has been previously proposed for low viscosity grade PAOs.^22^ By studying different collision energies, it has been concluded that the stability of the halogen adducts decreases when the analyte mass and number of repeating units decrease, and thus the smaller oligomers cannot be observed.^22,30^ Additionally, Nagy et al.^31^ reported faster fragmentation rates of chloride adducts to neutral species and free chloride ions for the short-chain polyisobutylene derivatives. The role of ion internal energy toward fragmentation has been discussed previously, since the dissociation energies for the chloride adducts are seemingly low.^32,33^ Therefore, it can be assumed that the small PS oligomers, consisting of 12 to 28 carbon atoms, easily dissociate into neutral species during ionization and thus are not observed in the spectrum.

On the other hand, PS oligomers with higher molecular weights are more stable and are therefore detected.

Also, the size of the halogen anion affected the obtained mass spectra because some smaller oligomers were not detected with increasing halogen size. For example, for PS1000, oligomers with the degree of polymerization (DP) of 6 to 14 were detected with CH_2_Cl_2_, whereas with CH_2_Br_2_ and CH_2_I_2_, the average DP was shifted to higher values. Similar trends were also observed with the other studied polystyrene standards. Additionally, the base peak intensity decreased in the order of Cl > Br > I, being 50-fold higher for Cl and 15-fold higher for Br as compared to I. The same trend was observed by Mendes Siqueira et al.^22^ with polyalphaolefins. Larger anions shifted the molecular weight distributions of PAO adducts to higher *m*/*z* values, and thus the authors concluded that the oligomer detection may be dependent on the halogen size. Signal-to-noise ratios were also affected by the halogen of choice, and CH_2_Cl_2_ produced the highest quality adduct ion spectra,^22^ similar to our findings. Dissociation enthalpies have been determined to be lower for larger halides, which would result in the decreasing intensity of adduct ions with increasing halogen anion size.^30^ This could also explain the detection of smaller oligomers with chloride but not with the other halogens.

### Polyethylene

**Figure 4:**
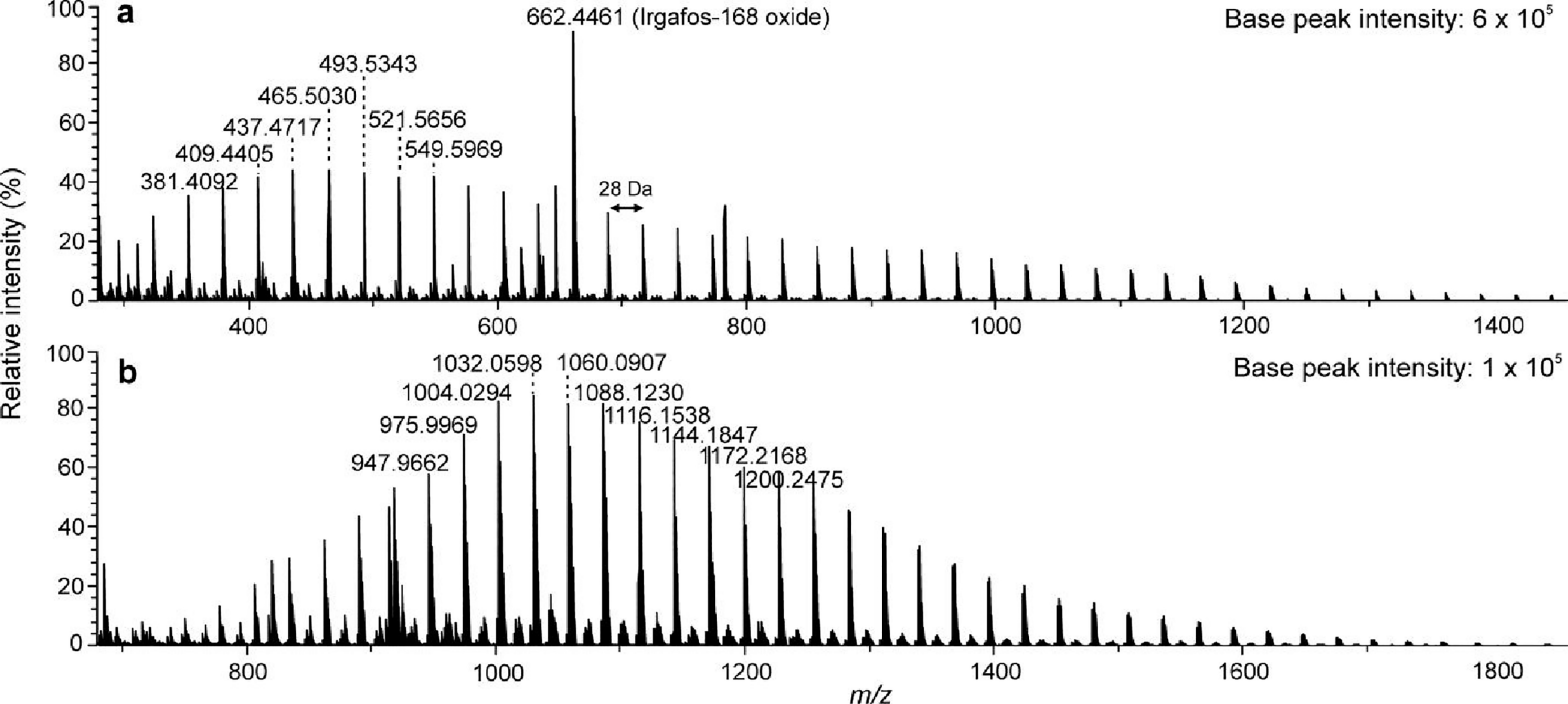
Mass spectra of PE4000 (a) in positive-ion mode APPI with toluene as a solvent and (b) on negative-ion mode APPI with toluene as a solvent and CH_2_Cl_2_ for halogen adduct formation. Peak at *m*/*z* 662.4461 is Irgafos-168 oxide.^14,34−36^ The absolute base peak intensities are reported for sensitivity comparison.

**Figure 5:**
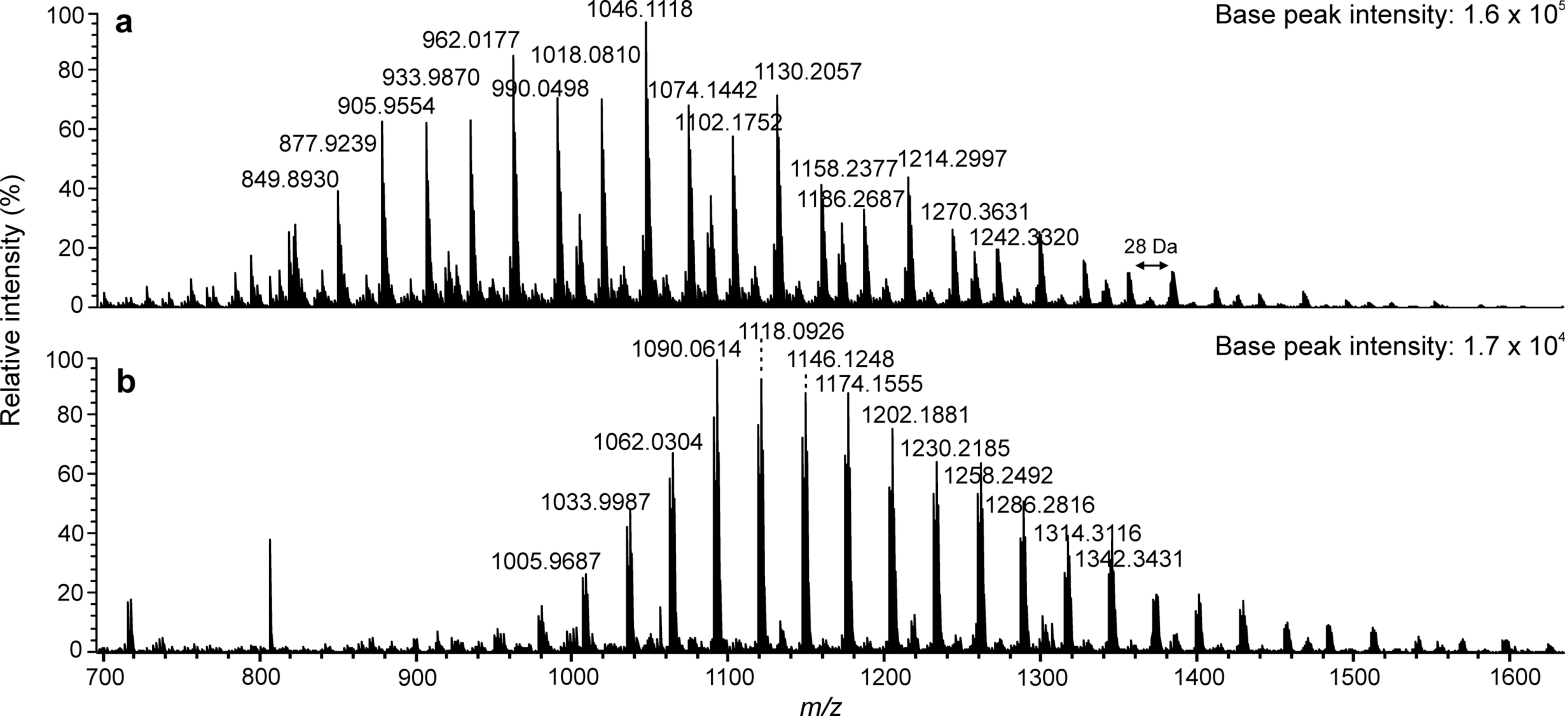
Negative-ion APPI-TOF mass spectra of PE800 with (a) CH_2_Cl_2_ or (b) CH_2_Br_2_ as the halogenated solvent and toluene as the dopant. The absolute base peak intensities are reported for sensitivity comparison.

Positive-ion APPI caused fragmentation and also formation of oxygenated species of PE oligomers, whereas negative-ion APPI with halogen attachment resulted in a more uniform, Gaussian-shaped ion distribution. The mass spectra of PE4000, measured in both ion modes, are presented in Figure 4. The calculated molecular weight values were *M*_n_ = 669 and *M*_w_ = 768 with (+) APPI and *M*_n_ = 1072 and *M*_w_ = 1100 with (−) APPI (CH_2_Cl_2_). The ions in the main series of (+) APPI spectra were identified as deprotonated oxygen adducts of polyethylene oligomers, [M – H + O]^+^, for both PE standards (Figures 4 and 5). The peak distributions were also skewed toward lower *m*/*z* values as compared to (−) APPI, indicating fragmentation of the polymer and underestimating the average molecular weight. In addition, signal-to-noise ratio was notably higher with halogen attachment for PE800, as the sample barely ionized with (+) APPI. The chloride attachment slightly overestimated the average MWs (*M*_n_ = 1007, *M*_w_ = 1025), probably because of the low mass discrimination, as only adducts with over 50 carbon atoms could be detected (*m*/*z* ≥ 737). Overall, there was less fragmentation and minimal oxygenation with PE standards as compared to (+) APPI, which is beneficial for structural characterization.

PE formed halogen adduct ions with chloride and bromide (Figure 5 for PE800), whereas iodide addition did not result in detected adducts. The mass difference between two adjacent ions was 28 Da, which corresponds to C_2_H_4_, the repeating unit of PE. The accurate masses of the ions corresponded to halogen adducts of saturated hydrocarbons, for example [C_72_H_146_ + Cl]^−^ for the PE800 ion at *m*/*z* 1046.1118 (Figure 5). The presence of halogen anion was further confirmed by comparing the experimental and calculated isotopic patterns, which correlated with each other. Additional peaks between the main ion series were identified as the halogen adduct ions of PE oligomers with odd numbers of carbon atoms and also some fragment ions based on accurate masses. When compared to PS, the observed PE spectra were “noisier” due to polymer fragmentation, which suggests that the aromatic polymer, such as polystyrene, results in more stable adduct ions than the purely aliphatic one, resulting in the “cleaner” mass spectra. Furthermore, some of the smaller oligomers were not detected with larger halogens. The PE oligomer distributions for chloride and bromide adducts had DP ≈ 25–54 and 31–54, respectively.

Halogen anion attachment also allowed detection of minor compositional variations in the polyethylene samples. Halogen-adduct ions in the main ion series of PE4000 were found to contain one oxygen atom (Figure 4b), contrary to PE800 (Figure 5). The oxygen atom most probably originates from the standard itself, since all PE800 ions were identified as adducts of pure hydrocarbon species. The experimental conditions for both polymers were identical. The oxygen atom could be due to hydroxyl-terminated PE oligomers, and halogen attachment has previously been successful in the end group analysis.^13,21^

Compositional differentiation was not possible with (+) APPI, where both PE polymers ionized as oxygenated species.

The results from PE800 corresponded well with previously studied PE standards with hydrogen end groups,^13^ although the second series of peaks was found to be 14 Da apart from the main series instead of 16 Da. This difference indicates that the second ion series is due to hydrocarbons with odd numbers of carbon atoms rather than the oxygen-containing oligomers in the study of Kéki et al.^13^ The higher molecular weight PE standards were also tested, but they did not form any adducts, probably due to low solubility and poor ionizability. The molecular weights of these PE standards were over 6000 Da, which is the highest mass that has been detected for various polymers by (−) APPI with halogen attachment.^12^

### Polyisoprene

**Figure 6:**
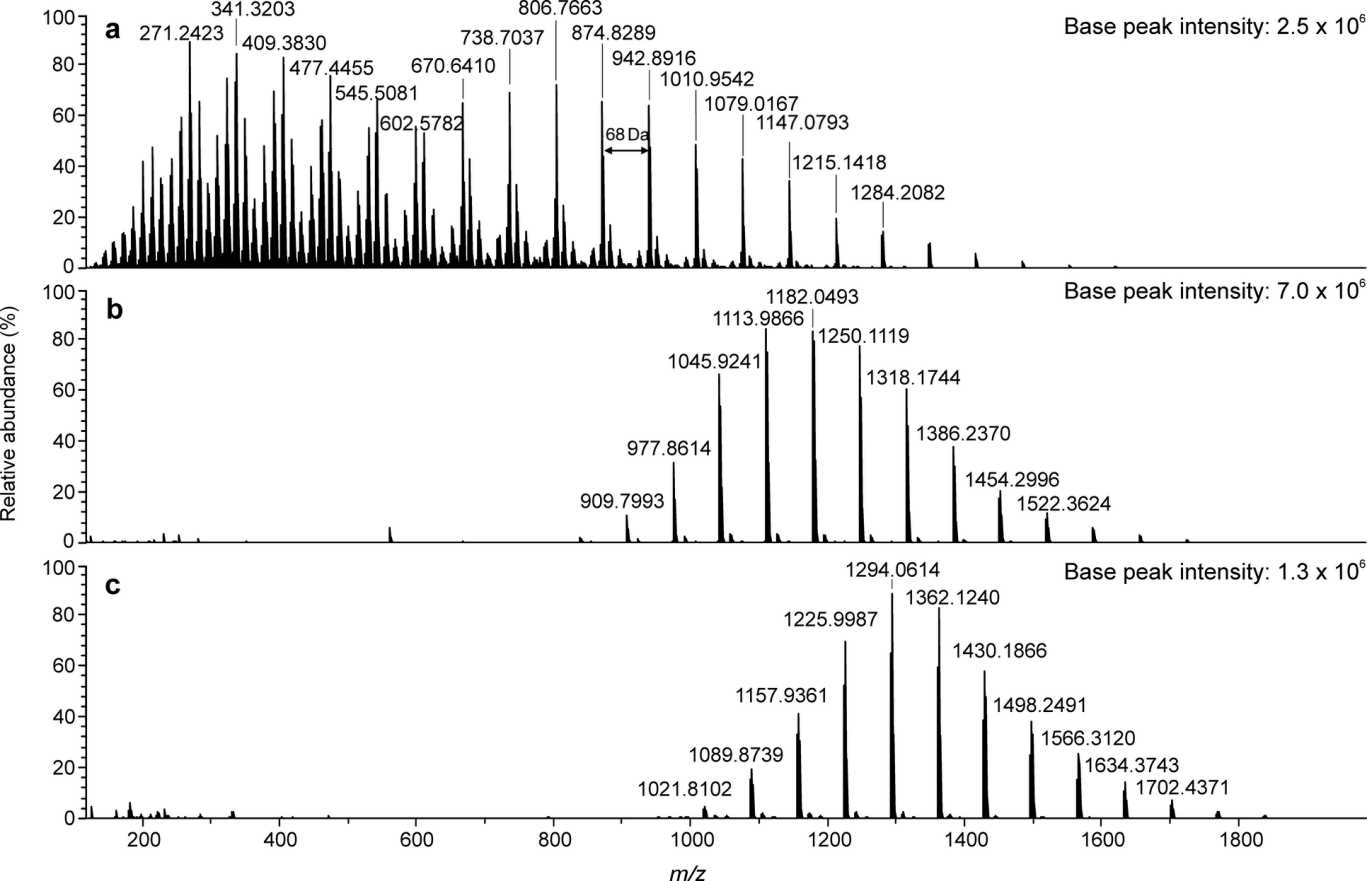
APPI-TOF mass spectra of PI1200 in (a) positive-ion mode with toluene as the dopant, (b) negative-ion mode with toluene as the dopant and CH_2_Cl_2_ as the halogen donor, and (c) negative-ion mode with toluene as the dopant and CH_2_Br_2_ as the halogen donor. The absolute base peak intensities are reported for sensitivity comparison.

Polyisoprene analysis with (+) APPI yielded a molecular weight distribution at lower *m*/*z* than expected, indicating fragmentation similar to the other studied polymers. Fragmentation shifted the average molecular weight values of PI1200 to M_n_ = 838 and *M*_w_ = 899, which are significantly lower than the expected values, or the values obtained with halogen attachment (*M*_n_ = 1161, *M*_w_ = 1179; Figure 6). In addition, multiple PI ion distributions were present, and they had a wide molecular weight range. These signals were identified to include both M^+•^ and [M – C_4_H_10_]^+^ ions of PI oligomers, among other fragment ions. The latter mentioned species would result from the cleavages of butyl end groups from the oligomers. On the contrary, (−) APPI with halogen attachment resulted in minimal fragmentation, and one narrow ion distribution of halogen adduct ions. Therefore, halogen adduct formation was advantageous also in the case of polyisoprene.

Polyisoprene halogen adducts were detected with chloride and bromide anions (Figure 6b and c for PI1200), while the iodide adducts had too low intensities for detection. The adjacent ions were 68 Da apart, corresponding to the repeating unit of PI (C_5_H_8_). The halogen adduct formation was confirmed again by accurate masses and comparison of the experimental and theoretical isotopic distributions. On the basis of the accurate masses, the main ion series consisted of polyisoprene halogen adducts, for example DP = 16 for the chloride adduct at *m*/*z* 1182.0493, consistent with the formula [C_84_H_138_ + Cl]^−^. A few additional peaks were also observed, indicating that some fragmentation occurred during ionization. Analogous to PS and PE, the smaller PI oligomers (DP = 11 and 12) were not detected with a larger bromide anion.

PI800 and PI1200 spectra (Figure 6 and Figures S2) differed slightly, particularly by the higher mass oligomers. The PI800 spectrum included more additional signals and ion distributions than PI1200, indicating that more fragmentation of the polymer had occurred. However, the calculated *M*_n_ of 1027 and *M*_w_ of 1040 overestimated the molecular weight, but this is likely because of the low halogen affinity, since the mass of PI800 is close to the smallest detected adducts. Additionally, the second ion distribution for PI800 with a relative intensity of ∼30% was detected 56 Da (C_4_H_8_) apart from the major ions. These species had higher intensities than the intact PI800 adduct ions at higher masses (*m*/*z* ≥ 1200). Mahmoud et al.^37^ found similar ion distribution to be present for the depolymerized PI, but the distribution was not observed in the PI1200 spectra, which included mostly intact PI oligomers. These species also dominated the intact oligomer adducts at low *m*/*z* region for PI800, unlike the other polyisoprene standard.

Polyisoprene has not been previously analyzed using halogen anion attachment, and the studies have traditionally been based on pyrolysis combined with gas chromatography-mass spectrometry (GC-MS). Direct insertion probe (DIP) combined with APCI and related atmospheric solids analysis probe (ASAP) have also been used to study depolymerized polyisoprene, which resulted in several ion distributions. These methods allowed the characterization of most pyrolysis species, and GC-MS also allowed identification of differences between the isomers,^37^ but they did not give information about the molecular weights of the original polymer samples. For this purpose, halogen adduct formation is applicable and a complementary method in polyisoprene analysis, since it resulted in the expected molecular weight distributions.

### Polypropylene

Polypropylene did not ionize well with (+) APPI, and thus no information on the polymer was obtained. The polymer ions had very low ion intensities, and they could be barely identified from the sample background. On the other hand, by (−) APPI with halogen attachment, clear distributions of halogen adduct ions of PP oligomers were detected, allowing identification of the repeating unit and the average molecular weights (*M*_n_ = 1138 and *M*_w_ = 1172, for chloride adducts).

**Figure 7:**
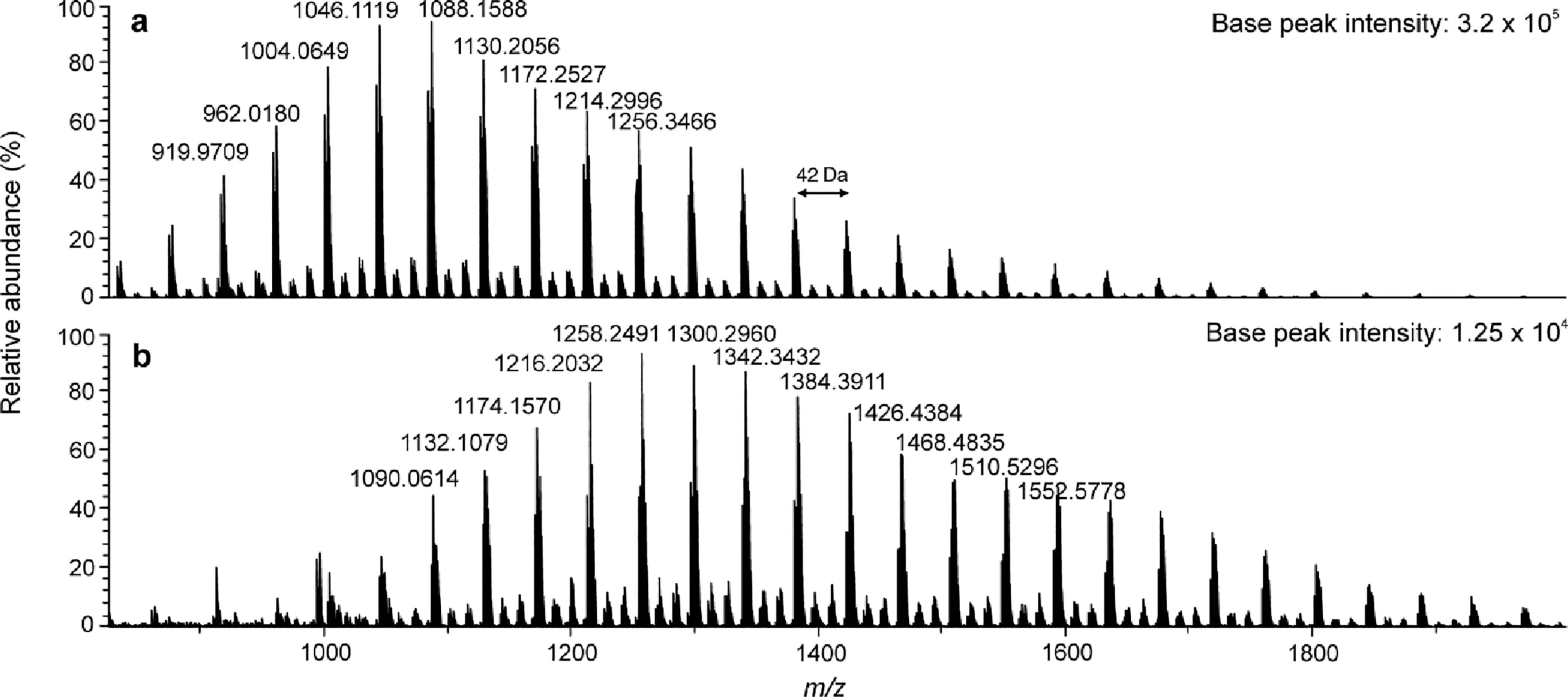
(−) APPI mass spectra for amorphous polypropylene with toluene as the dopant and (a) CH_2_Cl_2_ and (b) CH_2_Br_2_ as the halogen donors. The absolute base peak intensities are reported for sensitivity comparison.

Polypropylene formed halogen adduct ions in the presence of CH_2_Cl_2_ and CH_2_Br_2_ (Figure 7). The mass difference between the adjacent ions was 42 Da, which corresponds to the mass of the PP repeating unit of C_3_H_6_. The presence of halogen adducts was confirmed by accurate masses and comparing experimental and calculated isotopic patterns. The obtained mass spectra implied that some fragmentation occurred during ionization, since there were two additional ion series. These series were 14 Da apart from each other. Additionally, the halogen choice affected the observed oligomer distributions, and iodide did not form detectable adduct ions with PP. Overall, the adduct ion intensities were lower as compared to PS or PI, probably due to the lack of any ionizable functional groups, such as the aromatic rings or double bonds, in the saturated PP polymer.^15,16^

Polypropylene has previously been studied by pyrolysis-GC since direct analysis of the polymer is challenging due to its aliphatic nature.^14,15^ Halogen attachment would be a viable alternative for pyrolysis, as intact molecules could be analyzed for the approximate determination of their molecular weights. It would also simplify characterization of PP, since it produces only a single major ion distribution, contrary to multiple distributions, e.g., in ASAP-MS analysis.^14^ However, pyrolysis can also be advantageous since the fragments may help to reveal the fine structure of the polymer. For example, Farenc et al.^15^ characterized PP pyrolysis products using Fourier transform ion cyclotron resonance (FT-ICR) MS, combined with ion mobility separation, and these revealed small differences between isotactic and amorphous polypropylene.^15^ In this study, such differences were not observed as only the repeating unit, which is identical for both polypropylenes, could be determined.

### Hydrocarbons

Saturated hydrocarbons were also of interest in this study, especially since the results from polyethylene samples indicated that the saturated hydrocarbons could be analyzed via halogen attachment. Hydrocarbon analyses were started with (+) APCI, since the previous study by Tose et al.^24^ implied that larger hydrocarbons can be ionized via hydrogen abstraction to form nonclassical [M – H]^+^ ions in (+) APCI, by using small hydrocarbons, such as heptane or isooctane, as the solvent. However, application of this technique was not highly successful in this study. Hydrocarbons were ionized mainly as the oxygen adducts, [M – H + O]^+^, instead of [M – H]^+^ ions, which were barely observed in the spectra. Therefore, halogen attachment was chosen as a focus for saturated hydrocarbons due to its applicability with polymers.

On the basis of the obtained PE800 chloride adduct spectrum, hydrocarbons with fifty or more carbon atoms form halogen adducts, suggesting that *n*-alkanes of equal length should be ionizable. When pure *n*-alkanes were studied, pentacontane (C_50_H_102_) and hexacontane (C_60_H_122_) formed adduct ions with chloride, albeit at low intensity (Figure S3 and S4). However, chloride adducts were not detected for the shorter alkanes from tetradecane (C_14_H_30_) to tetracontane (C_40_H_82_). Various solvents and solution compositions were compared for these short alkanes, along with commercial hydrocarbon standard mixtures and instrument parameter changes, to limit the effects of solubility and experimental conditions on adduct formation. Despite the variations, none of the combinations resulted in the halogen adduct formation.

Short alkanes did not form any detectable chloride adducts, but it was found out that they formed oxygenated species in pure chlorinated solvents (CHCl_3_ or CH_2_Cl_2_) with (−) APPI. These species contained a varying amount of oxygen atoms and double bonds based on the accurate masses, and their identity was further confirmed by collision induced dissociation (CID) experiments, which revealed oxygenated hydrocarbon signals 14 Da apart. The formation of these species may be due to trace oxygen present in the APPI source, as even small amounts of oxygen can affect the ionization process.^19,38^ Pentacontane also formed oxygenated species in a pure chlorinated solvent, but halogen adduct ions appeared in toluene solution. This observation supports the proposed mechanism for halogen adduct formation, in which toluene (or other dopant) is needed for the reaction initiation.^12,13,20^

As proposed for small polystyrene oligomers, the lack of halogen adducts for shorter *n*-alkanes could possibly be explained by their low gas phase stabilities. Mendes Siqueira et al.^22^ did not observe any adducts for polyalphaolefins comprising oligomers with 30 carbon atoms, and they estimated that the species with less than 50 carbon atoms could not be detected. The results for the hydrocarbons in this study support this finding as no halogen adducts were detected for hydrocarbons with containing ≤ 50 carbon atoms. Additionally, the first chloride adducts in the obtained PE and PS spectra appeared at around *m*/*z* 700, corresponding approximately to C_50_, which would indicate a limit of detection at this mass range. Contrary to these results, Song et al.^19^ obtained chloride adducts for PEG methyl ether (*M*_n_ ≈ 750) with (−) APPI at a mass range approximately from 460 to 700 Da, which corresponds to only 19–27 carbon atoms. However, PEG methyl ether contains oxygen atoms which also contribute to the ionization^10^ and may result in the lower detection limit, and thus this result cannot be directly compared to pure hydrocarbons. Additionally, the detection of adduct ions can depend on instrument parameters, such as collision energy, and result in differences between different studies.^39^

Intact hydrocarbon ions, especially for longer *n*-alkanes, have rarely been detected with common mass spectrometry methods. Alkanes are chemically inert and do not contain heteroatoms or aromatic groups that would be easily ionizable, which limits their analysis.^40^ On the basis of our results, halogen attachment would seem to be applicable only above a certain mass limit, whereas Li et al.^29^ were able to ionize various intact hydrocarbons with nitrogen ion insertion. Ionizable hydrocarbons included lower molecular weight hydrocarbons, such as tetradecane (C_14_H_30_), which could not be ionized via chloride attachment. In addition, pentacontane and hexacontane were ionized with nitrogen ion insertion as well as halogen attachment. The intensities were found to be higher for longer alkanes, and this was explained by increased reactivity and stability of the nitrogen adduct.^29^ The same phenomenon seems to occur with halogen attachment, although the lower molecular weight adducts are not observed. Therefore, halogen adduct formation is not universal for hydrocarbon analysis, and it would need more research to assess the applicability for shorter hydrocarbon species.

## Conclusions

Negative-ion APPI with halogen anion attachment was evaluated as the potential ionization method for hydrocarbons and hydrocarbon-based polymers and compared to that of the conventional positive-ion APPI technique. With most samples, positive-ion APPI caused undesirable fragmentation of the polymers, and polyethylene was additionally found to form oxygenated species. These complicate identification of the original polymers, especially in the case of unknown samples. On the contrary, (−) APPI with halogen anion attachment allowed polymer characterization as halogen adduct ions with minimal fragmentation and oxygenation, and it was thus studied further with different types of hydrocarbon samples, using different halogen anions.

All the studied polymers ionized with the halogen attachment technique, but there were minor differences in the ionization efficiencies. The polymers containing double bonds, i.e., PS and PI, led to the more intense ion distributions than completely saturated polymers, PE and PP. However, halogen attachment tends to under- or overestimate average molecular weights due to fragmentation and/or low mass discrimination. Nevertheless, the halogen attachment was found to be a promising method for characterization of all studied polymers, even with polyolefins that are traditionally difficult to ionize due to their aliphatic nature.

The halogen anion used for the adduct formation also affected the intensity of the obtained adduct ions and their detection. With an increasing halogen anion size, some of the smaller oligomers for all polymers were not detected, and the obtained ion distribution was also altered. Additionally, signal-to-noise ratios decreased with an increasing halogen size, and iodide adducts were not detected for most of the studied polymers.

Lastly, hydrocarbon analysis with chloride attachment was conducted but halogen adducts were only detected for long-chain alkanes (i.e., C_50_ and C_60_). The shorter *n*-alkanes did not yield adduct ions, probably because of their low stabilities in the gas phase, leading to their decomposition. Thus, more research is needed to find a universal method for hydrocarbon analysis, although halogen attachment is applicable for longer aliphatic hydrocarbons and hydrocarbon-based polymers.

## Supporting Information Available

Additional polymer sample information and supplementary mass spectra (PDF)

## References

[ref1] Mohanan N.; Montazer Z.; Sharma P. K.; Levin D. B. Microbial and Enzymatic Degradation of Synthetic Plastics. Front. Microbiol. 2020, 11, 580709 10.3389/fmicb.2020.580709. 33324366 PMC7726165

[ref2] Kaushal J.; Khatri M.; Arya S. K. Recent Insight into Enzymatic Degradation of Plastics Prevalent in the Environment: A Mini - Review. Clean. Eng. Technol. 2021, 2, 100083 10.1016/j.clet.2021.100083.

[ref3] Ali S. S.; Elsamahy T.; Koutra E.; Kornaros M.; El-Sheekh M.; Abdelkarim E. A.; Zhu D.; Sun J. Degradation of Conventional Plastic Wastes in the Environment: A Review on Current Status of Knowledge and Future Perspectives of Disposal. Sci. Total Environ. 2021, 771, 144719 10.1016/j.scitotenv.2020.144719. 33548729

[ref4] PlasticsEurope: Enabling a sustainable future. Plastics - the Facts 2021: An analysis of European plastics production, demand and waste data. https://plasticseurope.org/knowledge-hub/plastics-the-facts-2021/ (accessed 2022-09-23).

[ref5] Mase C.; Maillard J. F.; Paupy B.; Farenc M.; Adam C.; Hubert-Roux M.; Afonso C.; Giusti P. Molecular Characterization of a Mixed Plastic Pyrolysis Oil from Municipal Wastes by Direct Infusion Fourier Transform Ion Cyclotron Resonance Mass Spectrometry. Energy Fuels 2021, 35, 14828–14837. 10.1021/acs.energyfuels.1c01678.

[ref6] Geyer R.; Jambeck J. R.; Law K. L. Production, Use, and Fate of All Plastics Ever Made. Sci. Adv. 2017, 3, e1700782 10.1126/sciadv.1700782. 28776036 PMC5517107

[ref7] Horton A. A. Plastic Pollution: When Do We Know Enough?. J. Hazard. Mater. 2022, 422, 126885 10.1016/j.jhazmat.2021.126885. 34418830

[ref8] Terrier P.; Desmazières B.; Tortajada J.; Buchmann W. APCI/APPI for Synthetic Polymer Analysis. Mass Spectrom. Rev. 2011, 30, 854–874. 10.1002/mas.20302. 21246594

[ref9] Hanton S. D. Mass Spectrometry of Polymers and Polymer Surfaces. Chem. Rev. 2001, 101, 527–569. 10.1021/cr9901081. 11712256

[ref10] Steinkoenig J.; Cecchini M. M.; Reale S.; Goldmann A. S.; Barner-Kowollik C. Supercharging Synthetic Polymers: Mass Spectrometric Access to Nonpolar Synthetic Polymers. Macromolecules 2017, 50, 8033–8041. 10.1021/acs.macromol.7b02018.

[ref11] Hart-Smith G.; Barner-Kowollik C. Contemporary Mass Spectrometry and the Analysis of Synthetic Polymers: Trends, Techniques and Untapped Potential. Macromol. Chem. Phys. 2010, 211, 1507–1529. 10.1002/macp.201000107.

[ref12] Desmazières B.; Legros V.; Giuliani A.; Buchmann W. Synthetic Oligomer Analysis Using Atmospheric Pressure Photoionization Mass Spectrometry at Different Photon Energies. Anal. Chim. Acta 2014, 808, 220–230. 10.1016/j.aca.2013.11.036. 24370106

[ref13] Kéki S.; Nagy L.; Kuki Á.; Zsuga M. A New Method for Mass Spectrometry of Polyethylene Waxes: The Chloride Ion Attachment Technique by Atmospheric Pressure Photoionization. Macromolecules 2008, 41 (11), 3772–3774. 10.1021/ma8005476.

[ref14] Barrère C.; Maire F.; Afonso C.; Giusti P. Atmospheric Solid Analysis Probe-Ion Mobility Mass Spectrometry of Polypropylene. Anal. Chem. 2012, 84, 9349–9354. 10.1021/ac302109q. 23043679

[ref15] Farenc M.; Witt M.; Craven K.; Barrère-Mangote C.; Afonso C.; Giusti P. Characterization of Polyolefin Pyrolysis Species Produced Under Ambient Conditions by Fourier Transform Ion Cyclotron Resonance Mass Spectrometry and Ion Mobility-Mass Spectrometry. J. Am. Soc. Mass Spectrom. 2017, 28, 507–514. 10.1007/s13361-016-1572-0. 28050872

[ref16] Jaber A. J.; Wilkins C. L. Hydrocarbon Polymer Analysis by External MALDI Fourier Transform and Reflectron Time of Flight Mass Spectrometry. J. Am. Soc. Mass Spectrom. 2005, 16, 2009–2016. 10.1016/j.jasms.2005.08.006. 16246576

[ref17] Kauppila T. J.; Kotiaho T.; Kostiainen R.; Bruins A. P. Negative Ion-Atmospheric Pressure Photoionization-Mass Spectrometry. J. Am. Soc. Mass Spectrom. 2004, 15, 203–211. 10.1016/j.jasms.2003.10.012. 14766288

[ref18] Kauppila T. J.; Syage J. A.; Benter T. Recent Developments in Atmospheric Pressure Photoionization-Mass Spectrometry. Mass Spectrom. Rev. 2017, 36, 423–449. 10.1002/mas.21477. 25988849

[ref19] Song L.; Wellman A. D.; Yao H.; Bartmess J. E. Negative Ion-Atmospheric Pressure Photoionization: Electron Capture, Dissociative Electron Capture, Proton Transfer, and Anion Attachment. J. Am. Soc. Mass Spectrom. 2007, 18, 1789–1798. 10.1016/j.jasms.2007.07.015. 17719234

[ref20] Kéki S.; Török J.; Nagy L.; Zsuga M. Atmospheric Pressure Photoionization Mass Spectrometry of Polyisobutylene Derivatives. J. Am. Soc. Mass Spectrom. 2008, 19, 656–665. 10.1016/j.jasms.2008.02.001. 18356077

[ref21] Kuckling D.; Zukowski M.; Rösener T.; Herres-Pawlis S. Atmospheric Pressure Photo-Ionization Mass Spectrometry for the Detection of Labile End Groups in Poly(Styrene). Eur. Polym. J. 2017, 90, 209–219. 10.1016/j.eurpolymj.2017.03.013.

[ref22] Mendes Siqueira A. L.; Beaumesnil M.; Hubert-Roux M.; Loutelier-Bourhis C.; Afonso C.; Pondaven S.; Bai Y.; Racaud A. Characterization of Polyalphaolefins Using Halogen Anion Attachment in Atmospheric Pressure Photoionization Coupled with Ion Mobility Spectrometry-Mass Spectrometry. Analyst 2018, 143, 3934–3940. 10.1039/C8AN00920A. 30051117

[ref23] Manheim J. M.; Milton J. R.; Zhang Y.; Kenttämaa H. I. Fragmentation of Saturated Hydrocarbons upon Atmospheric Pressure Chemical Ionization Is Caused by Proton-Transfer Reactions. Anal. Chem. 2020, 92, 8883–8892. 10.1021/acs.analchem.0c00681. 32453940

[ref24] Tose L. V; Cardoso F. M. R.; Fleming F. P.; Vicente M. A.; Silva S. R. C.; Aquije G. M. F. V.; Vaz B. G.; Romão W. Analyzes of Hydrocarbons by Atmosphere Pressure Chemical Ionization FT-ICR Mass Spectrometry Using Isooctane as Ionizing Reagent. Fuel 2015, 153, 346–354. 10.1016/j.fuel.2015.03.004.

[ref25] Ayrton S. T.; Jones R.; Douce D. S.; Morris M. R.; Cooks R. G. Uncatalyzed, Regioselective Oxidation of Saturated Hydrocarbons in an Ambient Corona Discharge. Angew. Chem., Int. Ed. 2018, 57, 769–773. 10.1002/anie.201711190. 29193616

[ref26] Hourani N.; Kuhnert N. High Molecular Weight Non-Polar Hydrocarbons as Pure Model Substances and in Motor Oil Samples Can Be Ionized without Fragmentation by Atmospheric Pressure Chemical Ionization Mass Spectrometry. Rapid Commun. Mass Spectrom. 2012, 26, 2365–2371. 10.1002/rcm.6338. 22956329

[ref27] Jin C.; Viidanoja J.; Li M.; Zhang Y.; Ikonen E.; Root A.; Romanczyk M.; Manheim J.; Dziekonski E.; Kenttämaa H. I. Comparison of Atmospheric Pressure Chemical Ionization and Field Ionization Mass Spectrometry for the Analysis of Large Saturated Hydrocarbons. Anal. Chem. 2016, 88, 10592–10598. 10.1021/acs.analchem.6b02789. 27700066

[ref28] Nyadong L.; Quinn J. P.; Hsu C. S.; Hendrickson C. L.; Rodgers R. P.; Marshall A. G. Atmospheric Pressure Laser-Induced Acoustic Desorption Chemical Ionization Mass Spectrometry for Analysis of Saturated Hydrocarbons. Anal. Chem. 2012, 84, 7131–7137. 10.1021/ac301307p. 22881221

[ref29] Li X.; Yan X.; Cooks R. G. Functionalization of Saturated Hydrocarbons Using Nitrogen Ion Insertion Reactions in Mass Spectrometry. Int. J. Mass Spectrom. 2017, 418, 79–85. 10.1016/j.ijms.2016.11.017.

[ref30] Nagy L.; Kuki Á.; Deák G.; Purgel M.; Vékony Á.; Zsuga M.; Kéki S. Gas-Phase Interaction of Anions with Polyisobutylenes: Collision-Induced Dissociation Study and Quantum Chemical Modeling. J. Phys. Chem. B 2016, 120, 9195–9203. 10.1021/acs.jpcb.6b05655. 27483334

[ref31] Nagy L.; Pálfi V.; Narmandakh M.; Kuki Á.; Nyíri A.; Iván B.; Zsuga M.; Kéki S. Dopant-Assisted Atmospheric Pressure Photoionization Mass Spectrometry of Polyisobutylene Derivatives Initiated by Mono- and Bifunctional Initiators. J. Am. Soc. Mass Spectrom. 2009, 20, 2342–2351. 10.1016/j.jasms.2009.08.025. 19819723

[ref32] Zhu J.; Cole R. B. Formation and Decompositions of Chloride Adduct Ions, [M + Cl]^−^, in Negative Ion Electrospray Ionization Mass Spectrometry. J. Am. Soc. Mass Spectrom. 2000, 11, 932–941. 10.1016/S1044-0305(00)00164-1. 11073256

[ref33] Nagy L.; Nagy T.; Deák G.; Kuki Á.; Purgel M.; Narmandakh M.; Iván B.; Zsuga M.; Kéki S. Can Nonpolar Polyisobutylenes Be Measured by Electrospray Ionization Mass Spectrometry? Anion-Attachment Proved to Be an Appropriate Method. J. Am. Soc. Mass Spectrom. 2016, 27, 432–442. 10.1007/s13361-015-1307-7. 26620530

[ref34] Fouyer K.; Lavastre O.; Rondeau D. Direct Monitoring of the Role Played by a Stabilizer in a Solid Sample of Polymer Using Direct Analysis in Real Time Mass Spectrometry: The Case of Irgafos 168 in Polyethylene. Anal. Chem. 2012, 84, 8642–8649. 10.1021/ac301759q. 22989312

[ref35] Hermabessiere L.; Receveur J.; Himber C.; Mazurais D.; Huvet A.; Lagarde F.; Lambert C.; Paul-Pont I.; Dehaut A.; Jezequel R.; Soudant P.; Duflos G. An Irgafos® 168 Story: When the Ubiquity of an Additive Prevents Studying Its Leaching from Plastics. Sci. Total Environ. 2020, 749, 141651 10.1016/j.scitotenv.2020.141651. 32836131

[ref36] Trimpin S.; Wijerathne K.; McEwen C. N. Rapid Methods of Polymer and Polymer Additives Identification: Multi-Sample Solvent-Free MALDI, Pyrolysis at Atmospheric Pressure, and Atmospheric Solids Analysis Probe Mass Spectrometry. Anal. Chim. Acta 2009, 654, 20–25. 10.1016/j.aca.2009.06.050. 19850163

[ref37] Mahmoud Z.; Bray F.; Hubert-Roux M.; Sablier M.; Afonso C.; Rolando C. Regio- and Stereo-Specific Chemical Depolymerization of High Molecular Weight Polybutadiene and Polyisoprene for Their Analysis by High-Resolution Fourier Transform Ion Cyclotron Resonance Mass Spectrometry: Comparison with Pyrolysis-Comprehensive Two-Dim Gas Chromatography/Mass Spectrometry, Atmospheric Solid Analysis Probe, Direct Inlet Probe-Atmospheric Pressure Chemical Ionization Mass Spectrometry, and Ion Mobility Spectrometry-Mass Spectrometry. Anal. Chem. 2020, 92, 15736–15744. 10.1021/acs.analchem.0c02650. 32897057

[ref38] Kauppila T. J.; Kuuranne T.; Meurer E. C.; Eberlin M. N.; Kotiaho T.; Kostiainen R. Atmospheric Pressure Photoionization Mass Spectrometry. Ionization Mechanism and the Effect of Solvent on the Ionization of Naphthalenes. Anal. Chem. 2002, 74, 5470–5479. 10.1021/ac025659x. 12433075

[ref39] Cai Y.; Cole R. B. Stabilization of Anionic Adducts in Negative Ion Electrospray Mass Spectrometry. Anal. Chem. 2002, 74, 985–991. 10.1021/ac0108818. 11925001

[ref40] Huba A. K.; Huba K.; Gardinali P. R. Understanding the Atmospheric Pressure Ionization of Petroleum Components: The Effects of Size, Structure, and Presence of Heteroatoms. Sci. Total Environ. 2016, 568, 1018–1025. 10.1016/j.scitotenv.2016.06.044. 27363346

