# Supplementary material for: Atmospheric Pressure Photoionization with Halogen Anion Attachment for Mass Spectrometric Analysis of Hydrocarbons and Hydrocarbon-Based Polymers
Source: J Am Soc Mass Spectrom. 2024 Nov 5;35(12):3147–56. doi: 10.1021/jasms.4c00331 (PMC11622225; doi:10.1021/jasms.4c00331)
Supplement: Supplementary file 1 — js4c00331_si_001.pdf [file js4c00331_si_001.pdf]

Supporting information for

Atmospheric pressure photoionization with halogen anion attachment for mass spectrometric analysis of hydrocarbons and hydrocarbon-based polymers

*Essi Rytkönen, Juha Rouvinen, Janne Jänis, and Marko Mäkinen**

Department of Chemistry, University of Eastern Finland, Joensuu, Finland

**Corresponding author: Department of Chemistry, University of Eastern Finland, P.O. Box 111, FI-80101 Joensuu, Finland. Email: marko.makinen@uef.fi*

Contents:

Table S1. Molar mass data for the studied polymer standards.

Figure S1. Negative-ion APPI spectra of PS1800 with CH_2X_2 (X = Cl, Br, I) as the halogen donor.

Figure S2. Positive- and negative-ion APPI-TOF mass spectra of PI800

Figure S3. Negative-ion APPI mass spectrum of hexacontane with CH_2Cl_2 as the halogen donor.

Figure S4. Calculated and experimental isotopic distributions for hexacontane chloride adduct.

Table S1. Molecular weight data of the studied polymers as provided by the polymer suppliers.

Sample	M_n (g/mol)	M_w (g/mol)	M_p (g/mol)	$Đ_M$
PS162	162	162	162	1.00
PS266	266	266	266	1.00
PS370	370	370	370	1.00
PS560	533	607	578	1.14
PS1000	846	953	890	1.13
PS1800	1730	1810	1810	1.05
PE800	720	840	770	1.17
PE4000	~1700	~4000	N.A.	
PI800	733	813	806	1.11
PI1200	1150	1250	1280	1.09
iPP	~5000	~12000	N.A.	
aPP	N.A.	N.A.	N.A.	

M_w = weight average molecular weight; M_n = number average molecular weight; M_p = peak molecular weight; $Đ_M$ = polydispersity; N.A. = not available.

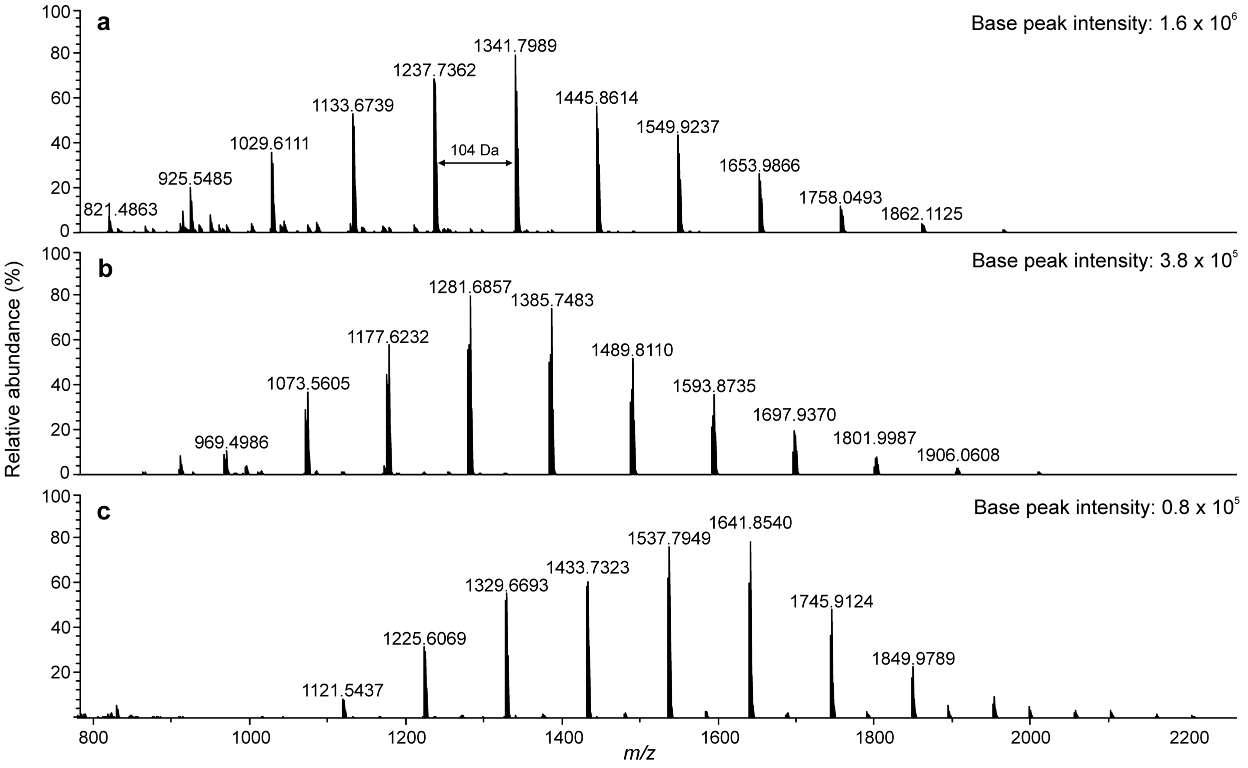

Figure S1. Negative-ion APPI-TOF mass spectra for PS1800 with toluene as the dopant and a) CH_2Cl_2, b) CH_2Br_2 and c) CH_2I_2 as the halogen donor.

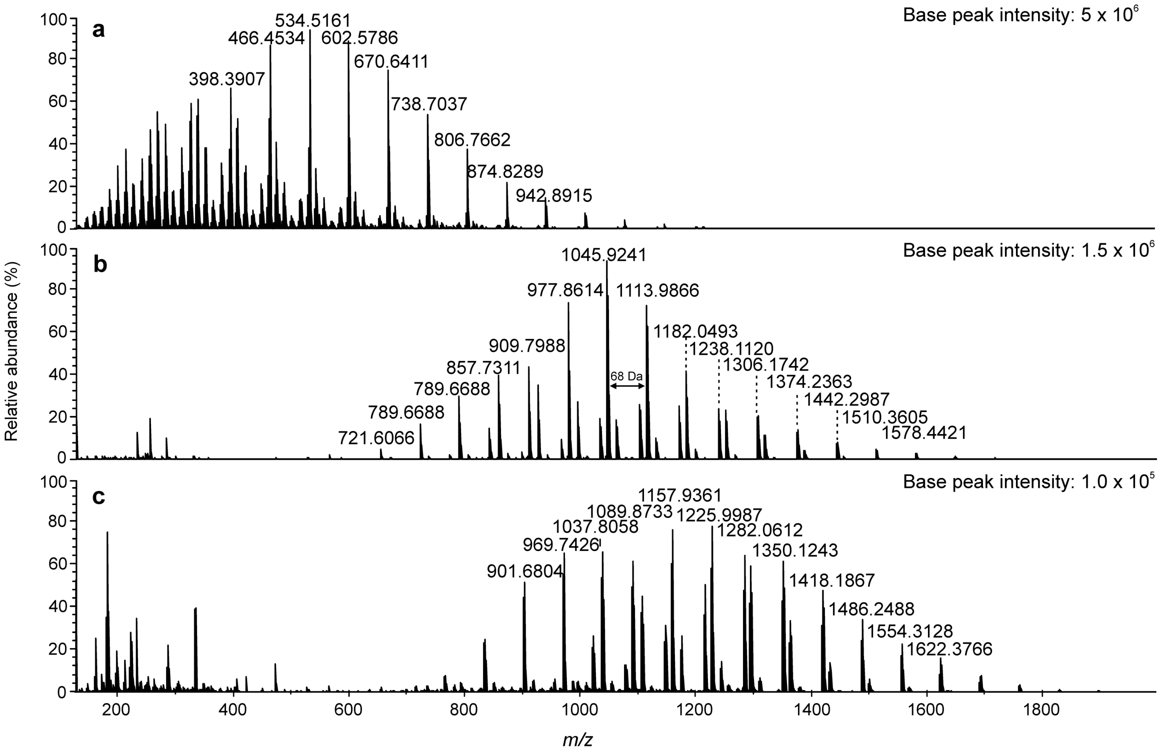

Figure S2. APPI-TOF mass spectra for PI800. a) Positive-ion mode with toluene as the dopant, b) negative-ion mode with CH_2Cl_2 as the halogen donor and toluene as the dopant, and c) negative-ion mode with CH_2Br_2 halogen donor and toluene as the dopant.

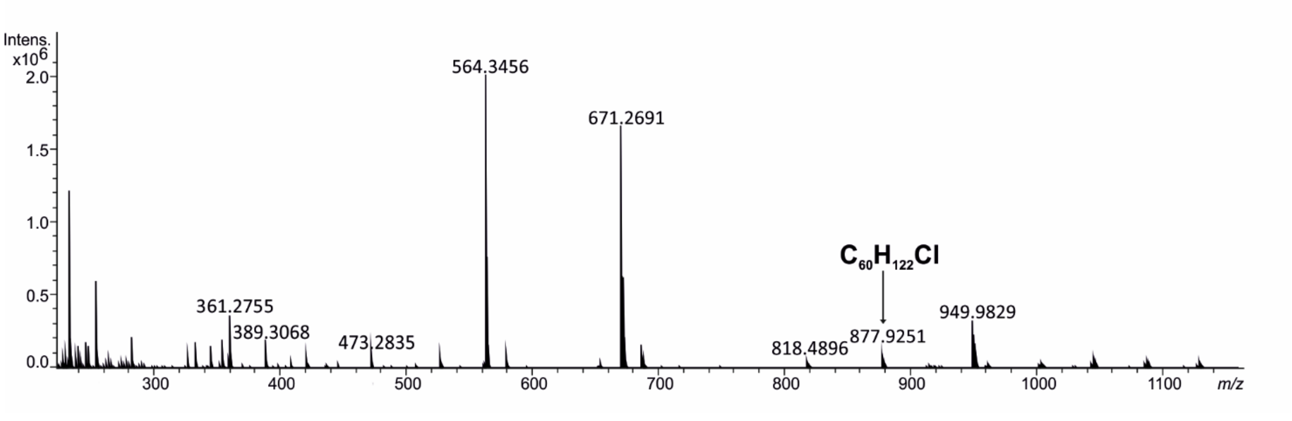

Figure S3. Negative-ion APPI-TOF mass spectrum of hexacontane with toluene as the dopant and CH_2Cl_2 as halogen donor. The chloride adduct $[C_{60}H_{122} + Cl]^-$ at *m/z* 877.9251 has been labeled. The other peaks represent unknown impurities.

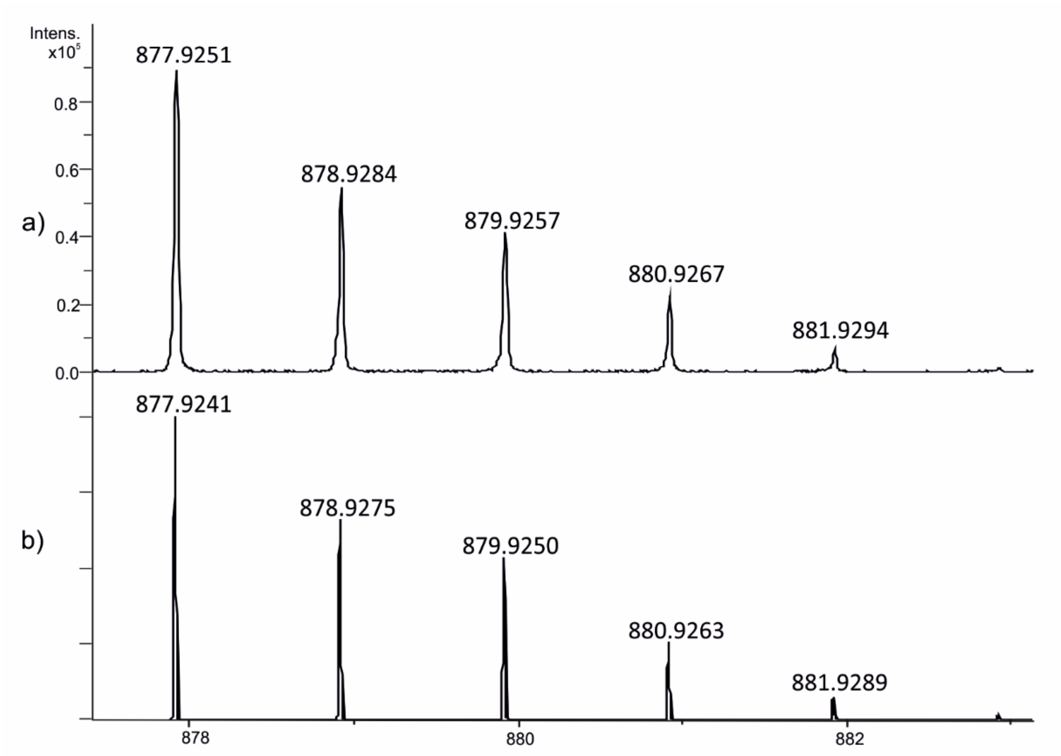

Figure S4. a) Experimental and b) theoretical isotopic distributions of hexacontane chloride adduct $[C_{60}H_{122}+Cl]^{-}$ at *m/z* 877.9 from hexacontane (–) APPI-TOF mass spectrum. Toluene was used as solvent and dopant, and dichloromethane was added to form chloride adduct ions.
